# SHP2 participates in decidualization by activating ERK to maintain normal nuclear localization of progesterone receptor

**DOI:** 10.1530/REP-22-0367

**Published:** 2023-06-09

**Authors:** Lin Chen, Weijie Zhao, Mengxiong Li, Yazhu Yang, Chengzi Tian, Dengyang Zhang, Zhiguang Chang, Yunzhe Zhang, Zhizhuang Joe Zhao, Yun Chen, Lin Ma

**Affiliations:** 1 Center for Reproductive Medicine, Seventh Affiliated Hospital, Sun Yat-sen University, Shenzhen, China; 2 Laboratory for Reproductive Immunology, NHC Key Lab of Reproduction Regulation (Shanghai Institute of Planned Parenthood Research), Shanghai Key Laboratory of Female Reproductive Endocrine Related Diseases, Hospital of Obstetrics and Gynecology, Fudan University Shanghai Medical College, Shanghai, China; 3 Department of Gynaecology, Seventh Affiliated Hospital, Sun Yat-sen University, Shenzhen, China; 4 Edmond H. Fischer Translational Medical Research Laboratory, Scientific Research Center, The Seventh Affiliated Hospital, Sun Yat-sen University, Shenzhen, China; 5 Faculty of Life Sciences and Medicine, Kings College London, London, United Kingdom; 6 Department of Pathology, University of Oklahoma Health Sciences Center, Oklahoma City, OK, United States

## Abstract

**In brief:**

The establishment and maintenance of embryo implantation and pregnancy require decidualization of endometrial stromal cells. This paper reveals that SHP2 ensures the correct subcellular localization of progesterone receptor, thereby safeguarding the process of decidualization.

**Abstract:**

Decidualization is the process of conversion of endometrial stromal cells into decidual stromal cells, which is caused by progesterone production that begins during the luteal phase of the menstrual cycle and then increases throughout pregnancy dedicated to support embryonic development. Decidualization deficiency is closely associated with various pregnancy complications, such as recurrent miscarriage (RM). Here, we reported that Src-homology-2-containing phospho-tyrosine phosphatase (SHP2), a key regulator in the signal transduction process downstream of various receptors, plays an indispensable role in decidualization. SHP2 expression was upregulated during decidualization. SHP2 inhibitor RMC-4550 and shRNA-mediated SHP2 reduction resulted in a decreased level of phosphorylation of ERK and aberrant cytoplasmic localization of progesterone receptor (PR), coinciding with reduced expression of IGFBP1 and various other target genes of decidualization. Solely inhibiting ERK activity recapitulated these observations. Administration of RMC-4550 led to decidualization deficiency and embryo absorption in mice. Moreover, reduced expression of SHP2 was detected in the decidua of RM patients. Our results revealed that SHP2 is key to PR's nuclear localization, thereby indispensable for decidualization and that reduced expression of SHP2 might be engaged in the pathogenesis of RM.

## Introduction

The establishment and maintenance of decidualization are vital for successful embryo implantation and the subsequent maintenance of a healthy pregnancy (Ng *et al.* 2020). During decidualization, the blastocyst adheres to the endometrial epithelium, and endometrial stromal cells (ESCs) around the blastocyst differentiate into round epithelioid cells, namely, specialized secretory decidual cells (Ochoa-Bernal & Fazleabas 2020). Moreover, ESCs undergo orderly proliferation and apoptosis, accompanied by the formation of polyploid cells and neovascularization (Gellersen *et al.* 2007). This morphological differentiation and high metabolic state prepare ESCs for subsequent embryo implantation (Zhang *et al.* 2013*b*). Abnormal decidualization allows embryo implantation without selectivity but cannot maintain pregnancy, which is closely related to abnormal tolerance of the maternal–fetal interface and recurrent miscarriage (RM) (Larsen *et al.* 2013). In-depth studies of the factors affecting the biological behavior of ESCs metaplasia will help to clarify the pathogenesis of RM and provide an important basis for finding more effective and accurate treatment methods.

RM is a common complication of pregnancy that affects the physical and mental health of pregnant women (Rai & Regan 2006). According to the guidelines of the American College of Obstetrics and Gynecology, RM is defined as two or more early abortions (Gynecologists 1995). Repeated embryo loss may lead to endometrial injury, endometritis, pelvic inflammatory disease, and infertility, which pose a serious threat to women’s reproductive health (Quenby *et al.* 2021). The common causes of RM include advanced maternal age, uterine abnormalities, immune disorders, hormone and metabolic disorders, environmental factors, and cytogenetic abnormalities (Toth *et al.* 2010). However, less than 50% of RM couples are diagnosed with a definite cause (Toth *et al.* 2010). Previous studies have shown that decidualization damage is related to the pathogenesis of RM (Larsen *et al.* 2013).

**Figure 1:**
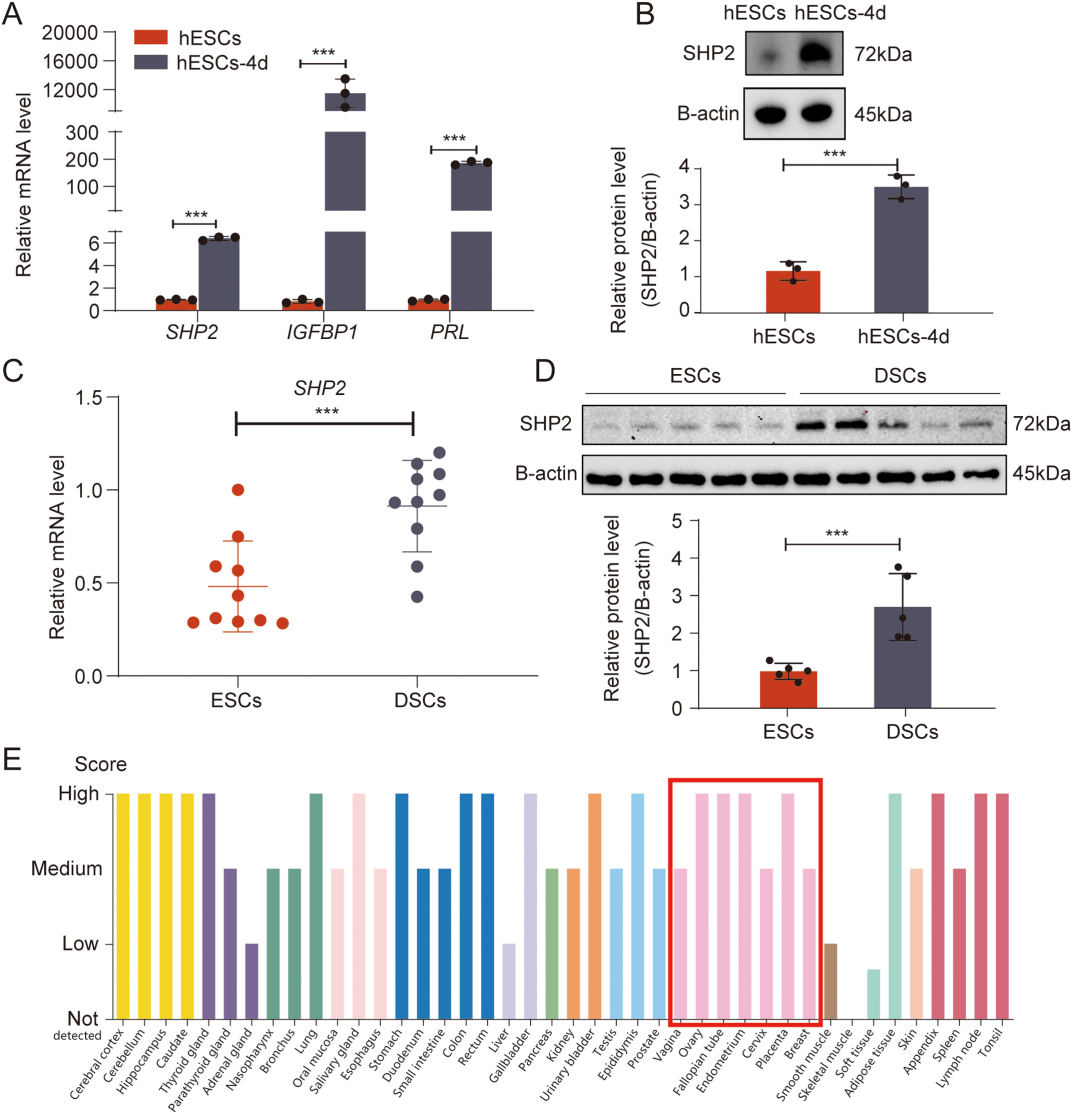
Artificially induced decidualization leads to the upregulation of SHP2 in hESCs. (A) Real-time PCR analysis of the transcriptional levels of *SHP2*, *IGFBP1,* and *PRL* in hESCs treated with MPA and db-cAMP for 4 days. Error bars denote the s.d. (*n* = 3). Data were calibrated to the most highly expressing sample in hESCs (red color). (B) Immunoblotting of the protein level of SHP2 in hESCs treated with MPA and db-cAMP for 4 days. Bar figures represent the ratios of densities (SHP2/B-actin). Error bars denote the s.d. (*n* = 3). (C) Real-time PCR analysis of the transcriptional levels of SHP2 in ESCs and DSCs. Error bars denote the s.d. (*n* = 10). Data were calibrated to the most highly expressing sample in ESCs (red color). (D) Immunoblotting of the protein level of SHP2 in ESCs and DSCs. Bar figures represent the ratios of densities (SHP2/B-actin). Error bars denote the s.d. (*n* = 5). (E) Bar graph of SHP2 expression across human tissues. Red box indicates SHP2 expression in female reproductive tissues. Data were obtained from the Human Protein Atlas. ^***^P < 0.001.

Src homology-2 domain-containing protein tyrosine phosphatase-2 (SHP2) is an important regulator of cell growth and differentiation and many signal transduction, which is widely expressed in various tissues (Feng 2007, Zhang *et al.* 2022*b*), especially in female reproductive tissues, as shown in Fig. 1E. Previous studies have shown that SHP2 is necessary for the Ras–ERK and PI3K–Akt pathways and plays a dynamic role in gametogenesis, embryo maturation, and embryonic development (Yuan *et al.* 2020). SHP2 can directly activate Ras by dephosphorylating p120Ras and can indirectly activate Ras by dephosphorylating KRAS, NRAS, and HRA through Src family kinases (SFKs) (Liu *et al.* 2021). SHP2 can also dephosphorylate substrate proteins important for activating the PI3K–Akt pathway, such as dephosphorizing Gab1 at the p85 binding site and terminating the Gab1/PI3K positive loop (Yu *et al.* 2002). SHP2 can also be regulated by Ras–ERK and PI3K–Akt pathway α-catenin dephosphorylation (Timmerman *et al.* 2012). SHP2 plays an important role in embryonic development and can induce trophoblast stem cell apoptosis by regulating FGF4-induced SFK–RAS–ERK signal transduction and inhibiting Bcl-2-like protein 11 (Bim) (Yang *et al.* 2006). Additionally, at embryo implantation, ESC nucleus SHP2 enhances its Tyr phosphorylation and dephosphorylates inhibitory Tyr in Src kinase, and ER-α recruits its target gene to activate ER-α (Ran *et al.* 2017). SHP2 is also associated with decidualization (Cheng *et al.* 2022), but the specific regulatory mechanism is not clear. At present, there is no study on the decidualization of the endometrium using SHP2 inhibitors, and the guiding role for RM patients is not clear. In the present study, we further explored the SHP2–ERK–PR signaling pathway, which is differentially expressed in the SHP2 inhibitor RMC-4550-exposed hESCs, RMC-4550-treated pregnant mice and RM tissues, to explore the roles of SHP2 in the regulation of this signaling pathway, to discover the mechanisms underlying the occurrence of miscarriage and decidual dysfunction caused by abortion and SHP2 deficiency and to provide a novel scientific and clinical understanding of the occurrence of miscarriage.

## Materials and methods

### Cell culture

Immortalized human ESCs (hESCs) were cultured in phenol red-free F12 medium containing 10% carbon-adsorbed serum (D-FBS), 100 U/mL penicillin, and 100 μg/mL streptomycin at 37°C in 5% CO_2_.

### Lentiviral shRNA transduction

Lentivirus-mediated SHP2 knockdown was performed as described previously (Stewart *et al.* 2003). Lentivirus‐mediated small hairpin RNA against SHP2 (sequence of sh-SHP2: CGCTAAGAGAACTTAAACTTT) and the non-target control (sequence of sh-Ctrl: CCTAAGGTTAAGTCGCCCTCG) were designed and constructed by OBio (Shanghai, China). Approximately 1 × 10^5^ cells/well were cultured in each of a 6-well plate, until a confluence of 70–80%. After replacing the cell culture medium with polystyrene-containing medium, sh-Ctrl or sh-SHP2 lentiviral particles were added and incubated for 6 hours. Subsequently, the medium was refreshed with fresh medium, and the cells were cultured for 72 hours before further treatments were conducted. SHP2 knockdown was confirmed by qPCR and western blotting.

### Cell treatment

To induce decidualization, hESCs were incubated with 1 μM MPA (Targetmol) and 0.5 mM db-cAMP (Targetmol). Subsequently, the SHP2 inhibitor RMC-4550 (Selleck) was added in the process of decidualization induction or hESCs knocked down SHP2 were used for decidualization induction. Decidualization was verified by the changes in cell morphology from a fibroblastic to a round shape, as observed with light microscopy, and five randomly selected microscopic fields in each group were used to calculate the relative ratio of round cells. To observe the translocation of PR, hESCs cells were treated with 2 μM P4 (Targetmol) and SHP2 inhibitor RMC-4550 (Selleck) or hESCs knocked down SHP2 were treated with 2 μM P4 (Targetmol). Concurrently, the MEK inhibitor trametinib (MCE) or ERK inhibitor U0126 (M Millipore) was added in the process of the translocation of PR. The PR translocation ratio was calculated as percentage of nuclear PR cells in five randomly selected microscopic fields.

### Tissue collection and primary cell isolation

All volunteers provided informed written consent, and the study received ethical approval from the Research Ethics Committee of the Seventh Affiliated Hospital of Sun Yat-sen University. Proliferative endometrial tissues were obtained from women with normal menstrual cycles via endometrial biopsy at the time of diagnostic hysteroscopy. Primary human ESCs were isolated according to previously described methods (Zhang *et al.* 2018). Briefly, endometrial tissues were fully washed with PBS and then minced in DMEM/F12. Minced tissues were digested with collagenase type IV for 1 h at 37°C. The suspension was filtered through 100-μm and 40-μm sterile filters and centrifuged at 300 ***g*** for 5 min. The supernatant was discarded, and the pellet was suspended in DMEM/F12 with 10% FBS. First-trimester (gestational age 6–10 weeks) human decidual tissues were obtained from clinically healthy controls (HCs, diagnosed as healthy pregnancies terminated for non-medical reasons) and RMs (diagnosed as recurrent spontaneous abortion, inclusion criteria included the presence of two or more consecutive abortions, and excluding parental and fetal genetic abnormalities, anatomic abnormalities, endocrine abnormalities, infection, etc.). Primary human decidual stromal cells (DSCs) were isolated according to previously described methods (Lv *et al.* 2019). Briefly, decidual tissues were obtained from HCs or RMs, and then the samples were washed in PBS and minced in DMEM/F12. The remaining tissues were embedded in paraffin, frozen, and transferred to liquid nitrogen for storage and further analysis.

### Human Protein Atlas

The Human Protein Atlas (www.proteinatlas.org) provides tissue and cell distribution information for all 24,000 human proteins through free public enquiries. We obtained SHP2 protein expression data in normal tissues for this study (Uhlen *et al.* 2005).

### SHP2 immunoprecipitation and PTP assays

SHP2-specific PTP assays were performed as described previously (Tzouvelekis *et al.* 2017). Briefly, hESCs treated with DMSO, 5 μM RMC-4550, or 10 μM RMC-4550 were lysed in RIPA buffer. Cell lysates were immunoprecipitated with an anti-SHP2 antibody or irrelevant control antibody. Immunoprecipitates were collected with Protein A agarose and washed extensively in RIPA buffer. The phosphatase activity protocol was adapted from McAvoy (McAvoy & Nairn 2010). Briefly, eluted proteins were added in triplicate in a 96‐well plate-containing PTP assay buffer (25 mM Tris-HCl, pH 7.5, 2 mM DTT, 1 mM EDTA) and 5 mM pNPP (New England Biolabs) in a final volume of 100 µL. The plate was incubated for 1 h at 37°C, and the reaction was stopped by the addition of NaOH for a final concentration of 1 N. The plate was then read at 405 nm in a BioTek SYNERGY H1. Assay was done in triplicate wells, and the experiment was repeated three times.

### Western blot analysis

Total protein extracts were prepared from homogenate tissues or cells cultured in RIPA buffer containing protease inhibitors and phosphatase inhibitors. The same amount of protein was separated by SDS‒PAGE before wet transfer to PVDF membranes. The membranes were blocked with 5% BSA for 1 h at room temperature and incubated with primary antibodies at 4°C overnight. Immunoreactivity was visualized by incubation with horseradish peroxidase-linked secondary antibodies and treatment with enhanced chemiluminescence reagents.

### Real-time quantitative PCR

Total RNA was extracted with the SteadyPure Universal RNA Extraction Kit (AG) according to the manufacturer’s instructions. The concentration and purity of the RNA were assessed using a spectrophotometer. Five hundred nanograms of total RNA was reverse transcribed using an Evo M-MLV RT Premix for qPCR Kit (AG), and the resulting cDNA was used as a template for qPCR. Each 20 μL qPCR system contained 2 μL cDNA, 1 μL forward primer (10 μM), 1 μL reverse primer (10 μM), 6 μL RNase-free ddH_2_O, and 10 μL SYBR Green Pro Taq HS Premix (AG). The primer sequences used are listed in Table 1. The samples were triplicated on the same plate. A standard curve was generated by using 10-fold serial dilutions (10^7^ to 10 copies), and standard curve correlation coefficients were greater than 0.98, with a mean coefficient of variation of 8%. The control wells had RNase-free ddH_2_O instead of cDNA and were routinely performed as negative controls. After incubation for 2 min at 50°C and a denaturation step of 2 min at 95°C, the samples were subjected to 44 cycles (15 s at 95°C, 1 min at 60°C), followed by the acquisition of the melting curve to verify the presence of a single amplicon. The qPCR was performed using Bio-Rad CFX96 Real-Time system. The primer efficiencies (E) were assessed through 10-fold standard dilution of cDNA (10^7^ to 10 copies) using the equation of E = 10^−1/slope^ − 1 and were determined to be between 1.9 and 2.0. The specificity of each assay was validated by melting curve analysis, agarose gel electrophoresis, and Sanger sequencing of the PCR products. Data were analyzed using the ΔCT method. Target gene expression was normalized to *ACTB* (human) or *Gapdh* (mouse) by taking the difference between CT values for target genes and *ACTB* (human) or *Gapdh* (mouse) (ΔCT value). These values were then calibrated to that of the most highly expressing sample in control (defined according to experimental designs) to give the ΔΔCT value. The fold target gene expression is given by the formula: 2^−ΔΔCT^. Experiments were performed three separate times. RT-qPCR was performed following the MIQE guidelines (Bustin *et al.* 2009).

**Table 1:** Primer sequences used for real-time PCR.

Gene	Forward	Reverse
*SHP2*	TGGTCCAGACAGAAGCACAG	GGCTCTGATCTCCACTCGTC
*IGFBP1*	TGCTGCAGAGGCAGGGAGCCC	AGGGATCCTCTTCCCATTCCA
*PRL*	CATCAACAGCTGCCACACTT	CGTTTGGTTTGCTCCTCAAT
*FOXO1*	CCGAGCTGCCAAGAAGAAAG	ATGCACATCCCCTTCTCCAA
*HOXA10*	TCACCAAGGCCAGCACATAG	TTAACTCAAGCTGCCTCGCC
*CEBPB*	AACTCTCTGCTTCTCCCTCTG	TGCGTCAGTCCCGTGTAC
*STAT3*	CTCCACTGGTCTATCTCTATC	ACTTGGTCTTCAGGTATGG
*HAND2*	ATGAGTCTGGTAGGTGGTTTTCC	CATACTCGGGGCTGTAGGACA
*PLZF*	GAGATCCTCTTCCACCGCAAT	CCGCATACAGCAGGTCATC
*HSD11B1*	TGGCTTATCATCTGGCGAAGA	AGGCAGTGGGATACCACCT
*BMP2*	AATGCAAGCAGGTGGGAAAG	GCTGTGTTCATCTTGGTGCA
*ACTB*	GGGAAATCGTGCGTGACATTAAG	TGTGTTGGCGTACAGGTCTTTG
*Dtprp*	TTATGGGTGCATGGATCACTCC	CCCACGTAAGGTCATCATGGAT
*Hand2*	AGATCAAGAAGACCGACGTGA	CTGTCCGGCCTTTGGTTTTC
*Hoxa10*	CCTAGAGATCAGCCGTAG	GACGTTGTCTGGAAGTTT
*Bmp2*	GAGAAAAGCGTCAAGCCAAAC	GGTGCCACGATCCAGTCATT
*Gapdh*	AGGTCGGTGTGAACGGATTTG	GGGGTCGTTGATGGCAACA

### Immunohistochemistry

All volunteers provided informed written consent, and the study received ethical approval from the research ethics committee of the Seventh Affiliated Hospital of Sun Yat-sen University. First-trimester (gestational age 6–10 weeks) human decidua was obtained from RM (inclusion criteria included the presence of two or more consecutive abortions, and excluding parental and fetal genetic abnormalities, anatomic abnormalities, endocrine abnormalities, infection, etc.) and normal pregnancies (terminated for nonmedical reasons). Decidual tissues were obtained immediately after surgery. Decidual specimens were fixed in 4% paraformaldehyde (PFA) and then embedded in paraffin. Tissue sections (5 μm) were deparaffinized, rehydrated, and incubated overnight at 4°C with primary antibodies. After washing with PBS, the sections were incubated with a horseradish peroxidase-conjugated secondary antibody; then, the reaction was developed with 3,3′-diaminobenzidine and counterstained with hematoxylin. The relative protein expression was quantified by ImagePro Plus version 6.0 software (Media Cybernetics Inc., Bethesda, MD, USA) and defined as follows: mean IOD = IOD sum/area sum. The IOD, as the positive-staining density, was measured as reported previously.

### Immunofluorescence staining

HESCs grown on coverslips were fixed with absolute methanol at room temperature for 20 min. After two washes with PBS, the cells were permeabilized with 0.5% Triton X-100 in 1× PBS for 30 min and blocked with PBS containing 3% BSA for 1 h at room temperature. The cells were then sequentially incubated with primary antibodies overnight at 4°C and with a fluorescein isothiocyanate-labeled secondary antibody for 1 h at room temperature. Immunofluorescence staining of human decidua tissue in frozen sections and mouse decidual tissue in the paraffin section was performed in the same steps. The sections were observed under a Leica fluorescence microscope. Information about the antibodies utilized is provided in Table 2. Images of IF were analyzed by Image-Pro Plus version 6.3. The number of positive cells was determined by using the count feature in the software. The red color is used to represent positive cells, and the blue color is used to represent the nucleus and thus indicate the total number of cells.

**Table 2:** Primary antibody used in the assay of western blotting (WB), immunofluorescence (IF), immunofluorescence (IF), immunohistochemistry (IHC), immunoprecipitation (IP).

Antibody	Cat. No.	RRID	Dilution				Company
			WB	IF	IHC	IP	
SHP2	3397	AB_2174959	1:1000	1:50	1:50	1:100	Cell Signaling Technology
IGFBP1	31025	AB_2798998	1:1000				Cell Signaling Technology
PRL	ab188229	AB_2921370	1:1000				Abcam
p-ERK	4370	AB_2315112	1:1000				Cell Signaling Technology
ERK	4695	AB_390779	1:1000				Cell Signaling Technology
B-actin	12620	AB_2797972	1:1000				Cell Signaling Technology
FOXO1	ab52857	AB_869817		1:100			Abcam
PR	AF6106	AB_2834993		1:100			Affinity Biosciences

Cat. No., catalog number; RRID, research resource identifier.

### Animal study

All animal experiments were conducted in accordance with the guidelines of the Institutional Animal Care and Use Committee of the Shenzhen Graduate School of Peking University, were approved by the Institutional Ethics Committee of the Shenzhen Graduate School of Peking University, and complied with the Guidelines for the Care and Application of Experimental Animals published by the National Institutes of Health. Animals were housed in specific pathogen-free facilities at the Peking University Shen Zhen Graduate School. Female C57BL/6 mice aged 6–8 weeks in estrus were bred with male C57BL/6 mice aged 8–10 weeks, and the day when vaginal plugging was observed was considered gestational day 1 (GD1). On GD5 and GD6, pregnant mice were anesthetized at 09:00 h and 21:00 h, and corn oil with and without RMC-4550 was injected into the abdominal cavity of each mouse. On GD7, mice were dissected, the uterus was weighed, the implantation sites were counted, and the embryo absorption rates were calculated. The absorbed embryos were identified by their smaller size and darker than the larger, viable, pink, healthy embryos. The embryo absorption rates were calculated as follows: (number of absorbed embryos/(total number of embryos)) × 100 (Wang *et al.* 2014, Wolfson *et al.* 2015). The first part of the was used to isolate mouse decidual stromal cells (mDSCs), and the second part of which was fixed with 4% PFA and then used to make paraffin sections, and the rest was transferred to liquid nitrogen for storage.

### HE staining in paraffin sections

The mouse uterus tissue samples were fixed in 4% PFA overnight at 4°C. After fixation, samples were washed with 70% ethanol. Washed samples were processed and embedded in paraffin. Hematoxylin and eosin (HE) staining was performed on 5-μm paraffin sections. Primary antibodies and dilutions used are listed in Table 2 below.

HE staining was conducted according to routine protocols (Liu *et al.* 2017). Briefly, put the slices into toluene I and toluene II for treatment, then soak them in different concentrations of alcohol, wash the alcohol with distilled water, and put the cleaned slices into HE staining solution (Beyotime) to dye the nucleus and cytoplasm, and finally dehydrate and seal the slices. The mounted slides were then photographed using the Leica DM4B system and KFBIO Digital Pathology Section 198 Scanner.

### Isolate mouse decidual stromal cells (mDSCs)

The mDSCs| were isolated as described previously (Li *et al.* 2007). Briefly, uterine horns were dissected and cut into 3- to 5-mm pieces. The uterine tissues were placed in HBSS containing dispase (Invitrogen) and pancreatin (Sigma) for 1 h at room temperature and then 10 min at 37°C. The supernatant was discarded to remove the endometrial epithelial clumps. The digested tissue was thoroughly washed with HBSS (Sigma). Subsequently, it was incubated in HBSS supplemented with collagenase I (Sigma) at a temperature of 37°C for a duration of 45 minutes. The resulting digested content was then passed through sterile filters with pore sizes of 70 μm and 40 μm. The stromal cells in the filtrate were pelleted, resuspended in DMEM-F12 medium containing 5% BSA, then centrifuged, and cell precipitates were used for subsequent experiments.

### Statistical analysis

Data are presented as the mean ± s.d. of at least three independent experiments. Statistical analyses were performed using GraphPad Prism 9 software. Differences between the two samples were conducted using an unpaired Student’s *t*-test when the data met a normal distribution, otherwise, the Mann–Whitney *U* test will be used. For comparisons of more than two groups, a one-way analysis of variance followed by a Tukey test was performed. *P* < 0.05 shows statistical significance.

## Results

### SHP2 is upregulated during decidualization

To further investigate the role of SHP2 in decidualization, we used hESCs to induce decidualization. Specifically, we used db-cAMP and MPA to induce decidualization of hESCs for 4 days and observed that the expression levels of SHP2 mRNA (Fig. 1A) and protein (Fig. 1B) were significantly increased compared with those in the control. Notably, the mRNA levels of endometrial decidualization biomarkers (IGFBP1 and PRL) in induced cells also increased significantly (Fig. 1A).

In addition, consistent with our data from hESCs, we found that the expression of SHP2 at the mRNA and protein levels was significantly upregulated in DSCs derived from decidual tissues of early pregnancy compared with ESCs derived from normal hyperplastic endometrium (Fig. 1C and D). Using data from the human protein atlas (Uhlen *et al.* 2015), we also found that SHP2 has endometrium-enriched gene expression (Fig. 1E). Overall, these results supported that SHP2 may be involved in decidualization.

### The SHP2 inhibitor RMC-4550 and knockdown of SHP2 attenuate decidualization

**Figure 2:**
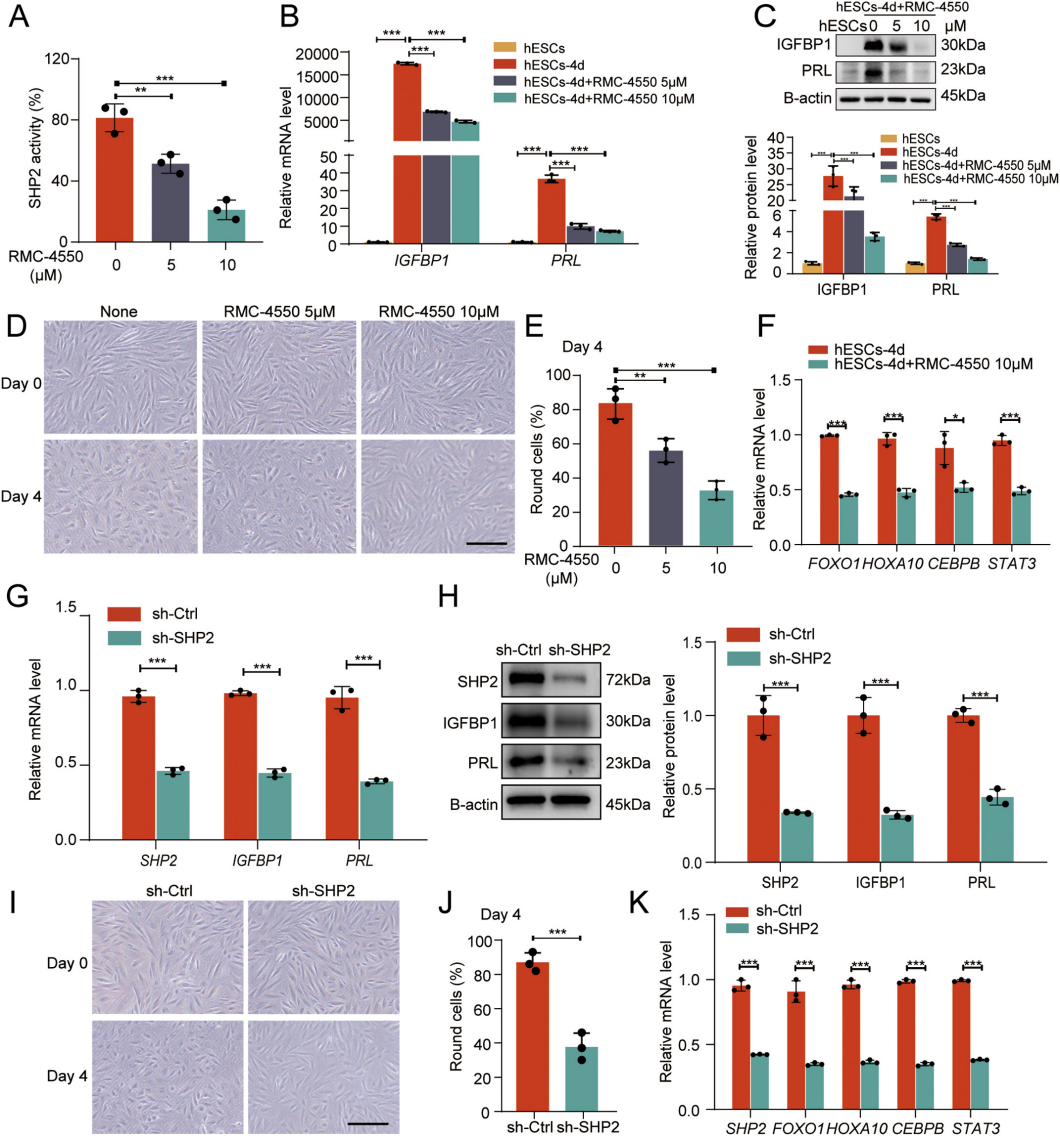
RMC-4550 and SHP2 knockdown inhibit artificially induced decidualization in hESCs. (A) SHP2 activity assay was performed. The hESCs were incubated for 1 h with or without RMC-4550. Error bars denote the s.d. (*n* = 3). (B) Real-time PCR analysis of the transcriptional levels of *IGFBP1* and *PRL* in hESCs with or without induced decidualization or treatment with RMC-4550. Error bars denote the s.d. (*n* = 3). Data were calibrated to the most highly expressing sample in hESCs (yellow color). (C) Immunoblotting of IGFBP1 and PRL protein levels in hESCs with or without induced decidualization or treatment with RMC-4550. Bar figures represent the ratios of densities (IGFBP1/B-actin and PRL/B-actin). Error bars denote the s.d. (*n* = 3). (D–E) The morphology of hESCs after decidualization with and without RMC-4550 treatment. The number of round cells from five different fields was counted and plotted as a histogram. Bar figures represent the ratios of round cells. Scale bars = 50 μm. (F) Real-time PCR analysis of the transcriptional levels of *FOXO1*, *HOXA10*, *CEBPB,* and *STAT3* in hESCs with or without induced decidualization or treatment with RMC-4550. Error bars denote the s.d. (*n* = 3). Data were calibrated to the most highly expressing sample in hESCs-4d (red color). (G) Real-time PCR analysis of the transcriptional levels of *SHP2*, *IGFBP1,* and *PRL* in hESCs, in which decidualization was induced for 4 days after transfection with sh-SHP2 or sh-Ctrl for 72 h. Error bars denote the s.d. (*n* = 3). Data were calibrated to the most highly expressing sample in sh-Ctrl (red color). (H) Immunoblotting of SHP2, IGFBP1, and PRL protein levels in hESCs, in which decidualization was induced for 4 days after transfection with sh-SHP2 or sh-Ctrl for 72 h. Bar figures represent the ratios of densities (SHP2/B-actin, IGFBP1/B-actin, and PRL/B-actin). Error bars denote the s.d. (*n* = 3). (I–J) The morphology of hESCs after decidualization, in which decidualization was induced for 4 days after transfection with sh-SHP2 or sh-Ctrl for 72 h. The number of round cells from five different fields were counted and plotted as histogram. Bar figures represent the ratios of round cells. Scale bars = 50 μm. K. Real-time PCR analysis of *SHP2* and the transcriptional levels of *FOXO1*, *HOXA10*, *CEBPB* and *STAT3* in hESCs, in which decidualization was induced for 4 days after transfection with sh-SHP2 or sh-Ctrl for 72 h. Error bars denote the s.d. (*n* = 3). Data were calibrated to the most highly expressing sample in sh-Ctrl (red color). ^*^P < 0.05, ^**^P < 0.01, and ^***^P < 0.001.

To examine how SHP2 affects the decidualization of the endometrium, we treated db-cAMP- and MPA-induced hESCs with an SHP2 inhibitor RMC-4550 to inhibit SHP2 function. The efficacy of this compound was confirmed *in vitro* using a PTP assay (Fig. 2A), which is consistent with previous reports (Nichols *et al.* 2018, Bracht *et al.* 2019). The SHP2 inhibitor RMC-4550 concentrations used were those previously reported in the literature (Lu *et al.* 2020, Quintana *et al.* 2020, Kano *et al.* 2021, Zhou *et al.* 2022). We noted that the expression of the decidualization markers IGFBP1 and PRL was significantly inhibited by additional RMC-4550 in decidualization-induced hESCs (Fig. 2B and C). We also found that the addition of RMC-4550 during decidualization resulted in morphological differences between db-cAMP and MPA-treated hESCs. Although control cells were enlarged and spherical on Day 4 of induction, the morphology of the additional RMC-4550-treated cells remained fibroblast-like (Fig. 2D and E), clearly indicating that inhibition of SHP2 blocks the extensive cellular morphological reprogramming that occurs during decidualization. Simultaneously, RMC-4550 significantly decreased the mRNA expression of *FOXO1*,* HOXA10*,* CEBPB,* and* STAT3*, four transcription factors that are known to be essential for decidualization (Fig. 2F). These results suggested that SHP2 may be involved in the regulation of decidualization of ESCs.

In order to further examine the effect of SHP2 on endometrial decidualization, we transfected hESCs with sh-SHP2 to knockdown SHP2 in db-cAMP and MPA-induced hESCs. Knockdown of SHP2 apparently attenuated the decidualization of hESCs, as verified by the reduced expression of IGFBP1 and PRL during induction (Fig. 2G and H). The sh-SHP2 induced group cells still retained a fibroblast-like shape, while the sh-Ctrl induced group cells changed into enlarged and spherical like morphology were observed (Fig. 2I and J). As critical transcriptional factors for decidualization,* FOXO1*,* HOXA10*,* CEBPB,* and* STAT3* were found to be downregulated during decidualization in sh-SHP2-treated cells (Fig. 2K). These results further suggest that SHP2 may participate in the regulation of decidualization of ESCs.

### RMC-4550 impairs decidualization in pregnant mice

**Figure 3:**
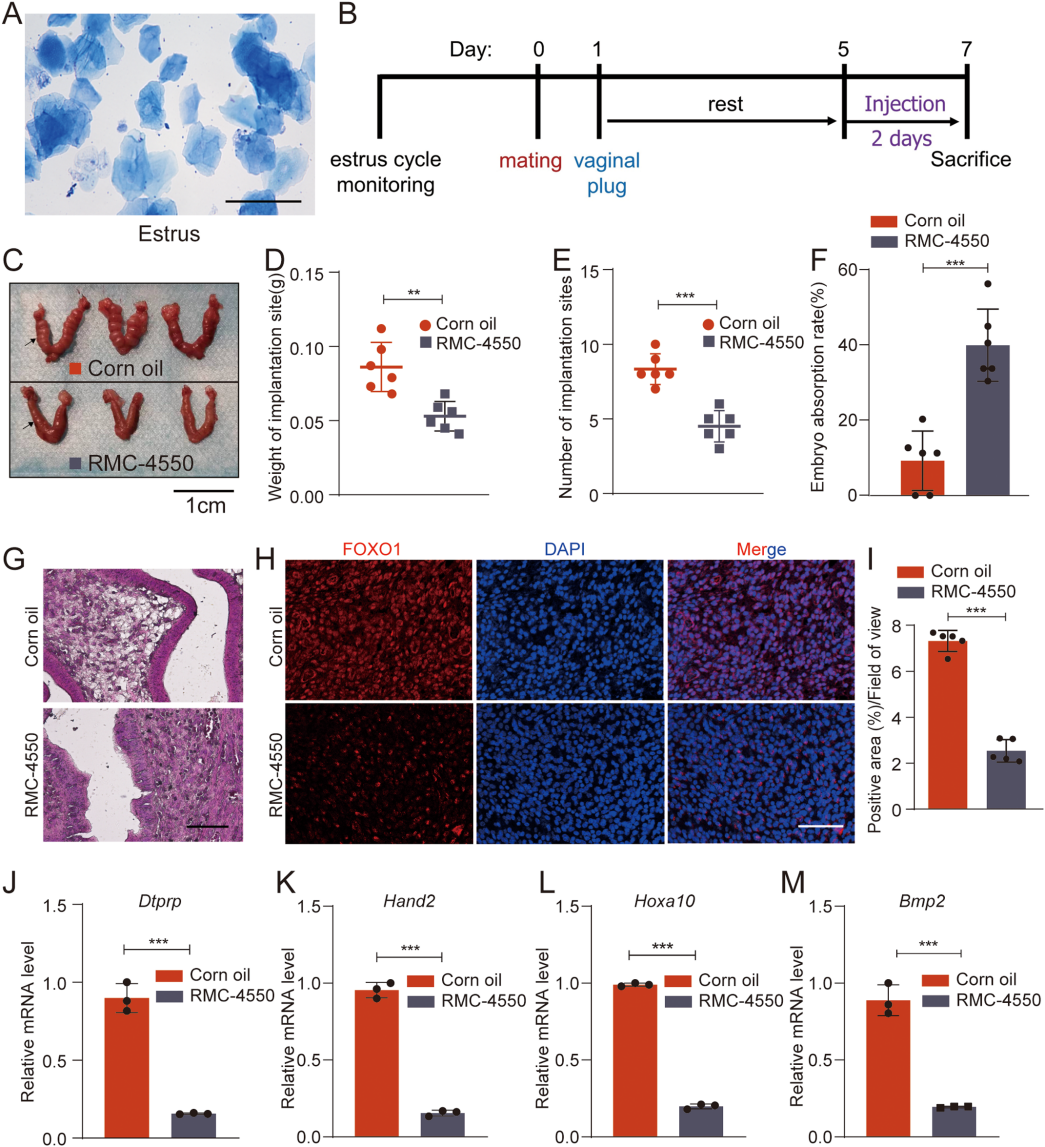
RMC-4550 inhibits decidualization of ESCs in pregnant mice. (A) A representative image of the smear of vaginal exfoliated cells stained with Giemsa from a mouse in estrus. Mice in estrus were selected for the *in vivo* study. Scale bars = 50 μm. (B) The schema of the animal study. (C) The morphology of uteri from RMC-4550-treated mice or corn oil-treated mice as controls. Scale bars = 1 cm. (D–F) The weights of uteri and the number of implantation sites and the embryo absorption rate from RMC-4550-treated mice or corn oil-treated mice as controls. Error bars denote the s.d. (*n* = 6). (G) HE staining in paraffin sections of mouse uterus tissues from corn oil-treated mice and RMC-4550-treated mice. Scale bars = 50 μm. (H–I) Immunofluorescent staining of FOXO1 in paraffin sections of mouse uterus tissues from corn oil-treated mice and RMC-4550-treated mice. Bar figures represent the ratios of positive area/field of view. Error bars denote the s.d. (*n* = 5). Scale bar=50 μm. (J–K) Real-time PCR analysis of the transcriptional levels of the mouse decidualization marker *Dtprp* and mouse PR downstream target genes *Hand2*, *Hoxa10,* and *Bmp2* in mDSCs from mice treated with RMC-4550 or corn oil as controls. Error bars denote the s.d. (*n* = 3). Data were calibrated to the most highly expressing sample in corn oil (red color). ^**^P < 0.01, ^***^P < 0.001.

We further verified the roles of SHP2 in decidualization in a pregnant mouse model. Mice in estrus (Fig. 3A) were selected for the experiment. The day when the vaginal plug was seen was recorded as GD1 of pregnancy. On GD5 and GD6 of pregnancy, RMC-4550 dissolved in corn oil was injected into the abdominal cavity of mice twice a day, and normal corn oil was injected into control mice (Fig. 3B). We dissected and weighed the mice on GD7 of pregnancy and found that RMC-4550 significantly reduced the decidualization of mice (Fig. 3C), the weights of the RMC-4550-treated mouse uteri (Fig. 3D) and the number of implantation sites (Fig. 3E) were significantly lower than those of the control group. Whereas, intraperitoneal RMC-4550 injection increased the embryo absorption rate (Fig. 3F). The histomorphology of the uterus was evaluated by HE staining (Fig. 3G). HE staining showed that the columnar epithelial structure of the decidua in the corn oil injection control group covered the surface of the uterine cavity completely and stromal cells in the decidua were rich in white lipid droplets, while the columnar epithelial structure was disorganized and stromal cells in the RMC-4550 treatment group contained less lipid droplets. In addition, this was confirmed by immunofluorescence staining of the mouse uterus sections with FOXO1, a known decidualization marker, as we observed a decreased expression of FOXO1 in the RMC-4550 treatment group (Fig. 3H and I). And the RMC-4550-treated mouse decidualization marker *Dtprp* was also significantly reduced (Fig. 3J). Furthermore, we also found that compared with the control group, the RMC-4550 group also exhibited decreased mRNA expression levels of *Hand2* (Fig. 3K), *Hoxa10* (Fig. 3L) and *Bmp2* (Fig. 3M), the downstream target genes of mouse uterine progesterone signaling. Altogether, these results demonstrated that SHP2 may be involved in the regulation of decidualization of the mouse endometrium and may play an important role in the maintenance of PR function.

### RMC-4550 impairs nucleocytoplasmic translocation of progesterone receptor (PR) and decreases PR target genes

**Figure 4:**
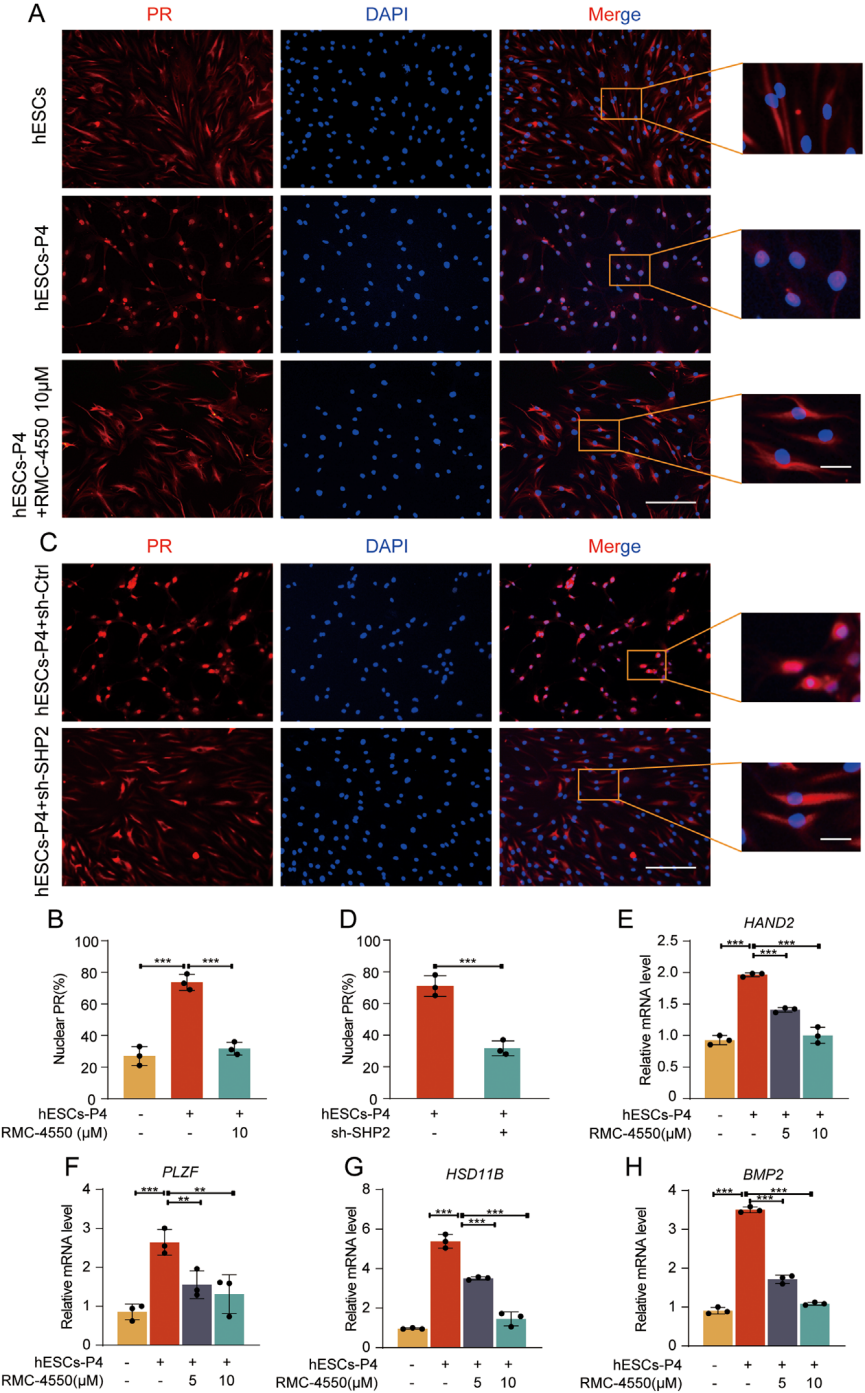
RMC-4550 inhibits the nuclear translocation of PR and the expression of PR target genes in hESCs treated with P4. (A–B) Immunofluorescent staining of PR (red) in hESCs stimulated with P4 (2  μM) with or without RMC-4550 treatment for 1 h. Nuclei were stained with DAPI (blue). The number of nuclear PR cells from five different fields was counted and plotted as a histogram. Bar figures represent the ratios of nuclear PR. Scale bar = 50 µm; 10 μm (magnified graphs). (C–D) Immunofluorescent staining of PR (red) in hESCs, in which stimulated with P4 (2 μM) for 1 h, after transfection with sh-SHP2 or sh-Ctrl for 72 h. Nuclei were stained with DAPI (blue). The number of nuclear PR cells from five different fields was counted and plotted as a histogram. Bar figures represent the ratios of nuclear PR. Scale bar = 50 µm; 10 μm (magnified graphs). (E–H) Real-time PCR analysis of the transcriptional levels of the PR target genes *HAND2*, *PLZF*, *HSD11B,* and *BMP2* in hESCs treated with or without P4 with or without RMC-4550. Error bars denote the s.d. (*n* = 3). Data were calibrated to the most highly expressing sample in blank control (yellow color). ^**^P < 0.01, ^***^P < 0.001.

Decidual tissue maintains pregnancy mainly through the function of PR, in which PR plays important roles during the process of ESCs decidualization. To explore the effect of SHP2 on the function of PR, hESCs and shRNA-mediated SHP2 reduction of hESCs were stimulated with P4 and/or RMC-4550. The results demonstrated that nucleocytoplasmic translocation of PR was both blocked in RMC-4550-treated cells (Fig. 4A and B) and in shRNA-mediated SHP2 reduction of hESCs (Fig. 4C and D), which indicated that SHP2 may be involved in the functional regulation of PR. In addition, we also observed a significant decrease in the mRNA expression of PR downstream target genes, such as *HAND2* (Fig. 4E), *PLZF* (Fig. 4F), *HSD11B* (Fig. 4G), and *BMP2* (Fig. 4H), in induced cells with additional RMC-4550 treatment. These data implied that SHP2 may be involved in maintaining the normal function of PR.

### RMC-4550 inhibits the activation of ERK, which is required for the nuclear localization of PR in P4-treated hESCs

**Figure 5:**
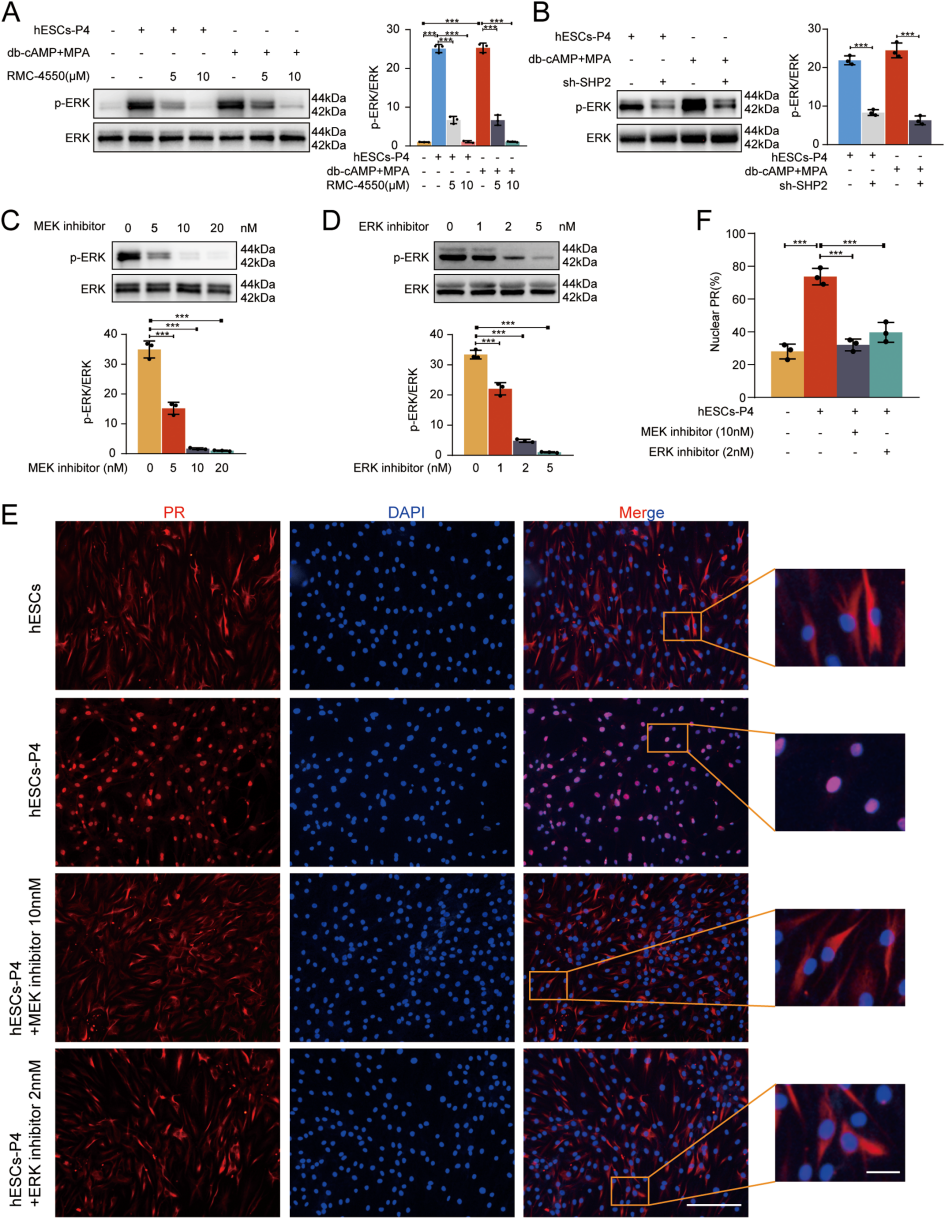
RMC-4550 or SHP2 knockdown inhibits the activation of ERK, which is required for nuclear localization of PR in P4-treated hESCs. (A–B) Immunoblotting of the phosphorylation levels of ERK and total ERK in hESCs stimulated with or without P4 /db-cAMP+MPA and with or without RMC-4550 treatment/SHP2 knockdown. Bar figures represent the ratios of densities (pERK/ERK). Error bars denote the s.d. (*n* = 3). (C–D) Immunoblotting of the phosphorylation levels of ERK and total ERK in hESCs stimulated with P4 and with or without MEK inhibitor trametinib/ERK inhibitor U0126 treatment. Bar figures represent the ratios of densities (pERK/ERK). Error bars denote the s.d. (*n* = 3). (E–F) Immunofluorescent staining of PR (red) in hESCs stimulated with P4 and with or without MEK inhibitor trametinib or ERK inhibitor U0126 treatment. Nuclei were stained with DAPI (blue). The number of nuclear PR cells from five different fields was counted and plotted as histogram. Bar figures represent the ratios of nuclear PR. Scale bar = 50 µm; 10 μm (magnified graphs). ^***^P < 0.001.

The function of PR is realized through the translocation of PR from the cytoplasm to the nucleus, where it then participates in the transcriptional regulation of target genes. To further explore the regulatory mechanism of SHP2 on PR function, the SHP2 inhibitor RMC-4550-treated hESCs or shRNA-mediated SHP2 reduction of hESCs were treated with P4 or db-cAMP and MPA. Adding RMC-4550 or shRNA-mediated SHP2 reduction reduced the level of ERK phosphorylation (Fig. 5A and B). Simultaneously, we observed that the MEK inhibitor trametinib or ERK inhibitor U0126 could also reduce the level of ERK phosphorylation (Fig. 5C and D) and block the nuclear cytoplasmic translocation of PR (Fig. 5E and F) in hESCs treated with P4. The MEK inhibitor trametinib (Zhou *et al.* 2018, Ma *et al.* 2019, Tang *et al.* 2021) and ERK inhibitor U0126 (Liu *et al.* 2020, Ranall *et al.* 2010, Zhang *et al.* 2013*a*) concentrations used were those previously reported in the literature. These data suggested that SHP2 may be involved in maintaining the normal function of PR by regulating the activation of ERK. Taking these data together, we suggest that the upregulation of SHP2 is part of the decidualization process and that the upregulation of SHP2 is involved in decidualization by maintaining normal nuclear localization of PR.

### SHP2 expression is decreased in the decidual tissues of RM patients

**Figure 6:**
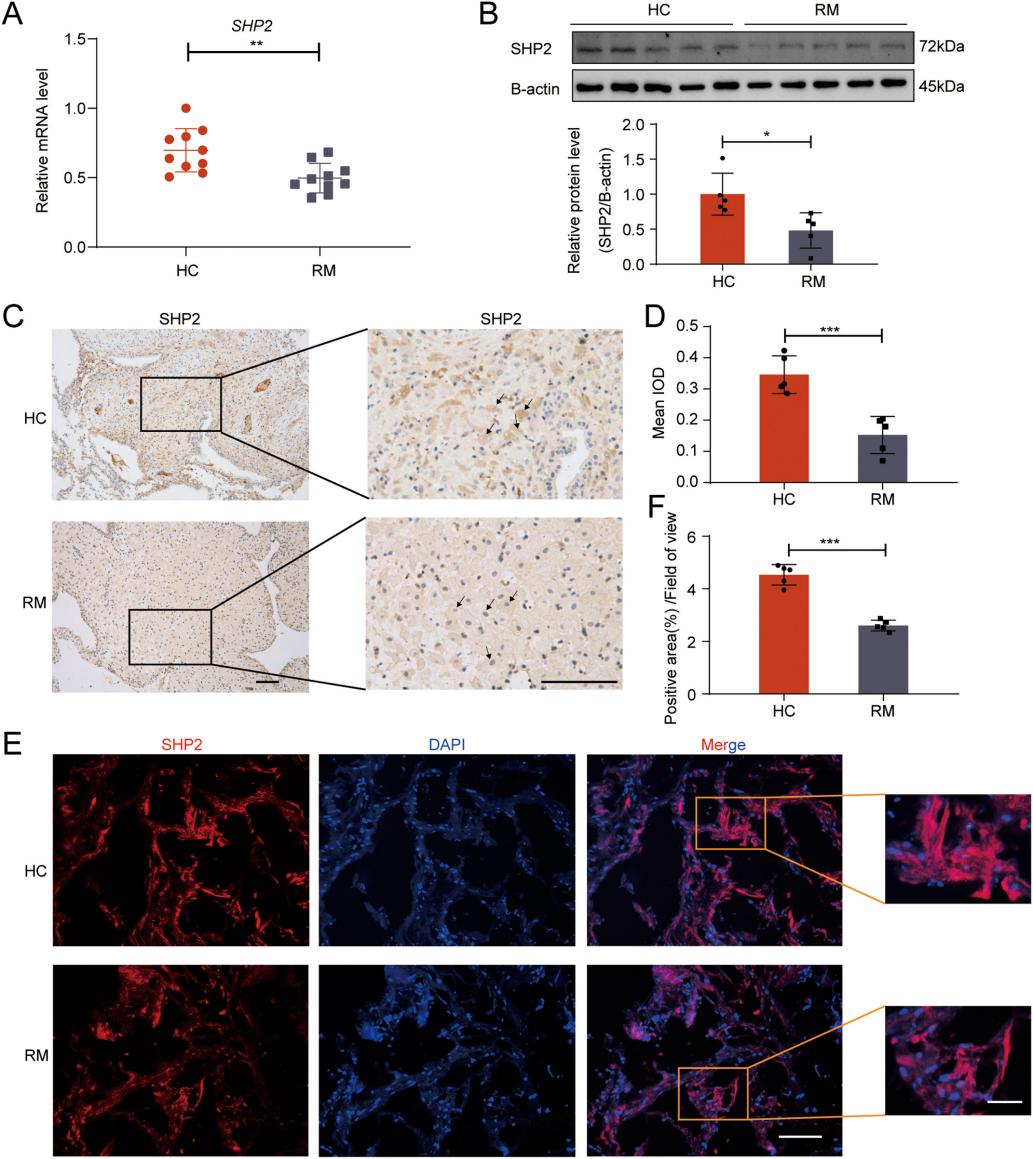
SHP2 is downregulated in the decidua of patients with RM. (A) Real-time PCR analysis of the transcriptional level of *SHP2* in decidual tissues from donors with normal pregnancies and patients with RM. Error bars denote the s.d. (*n* = 10). Data were calibrated to the most highly expressing sample in HC (red color). (B) Immunoblotting of the protein level of SHP2 in decidual tissues from donors with normal pregnancies and patients with RM. Bar figures represent the ratios of densities (SHP2/B-actin). Error bars denote the s.d. (*n* = 5). (C–D) Immunohistochemistry staining of SHP2 in paraffin sections of decidual tissues from donors with normal pregnancies and patients with RM. Brown staining represents the target protein. Arrowheads indicate the decidual stromal cells. Bar figures represent IOD. Error bars denote the s.d. (*n* = 5). Scale bar = 50 μm. (E–F) Immunofluorescent staining of SHP2 in frozen sections of decidual tissues from donors with normal pregnancies and patients with RM. Bar figures represent the ratios of positive area/field of view. Error bars denote the s.d. (*n* = 5). Scale bar=50 μm;10 μm (magnified graphs). ^*^P < 0.05, ^**^P < 0.01, ^***^P < 0.001.

We further detected the expression of SHP2 in the decidua of RM. We first performed real-time PCR and western blot analysis of decidual tissues from early pregnancy in patients with RM and HC. SHP2 expression was downregulated significantly in the decidual tissue of patients with RM (Fig. 6A and B). Furthermore, immunohistochemical analysis of paraffin-embedded decidual tissue (Fig. 6C and D) and immunofluorescent staining analysis of frozen sections of decidual tissue (Fig. 6E and F) also showed that the expression of SHP2 in RM decidual tissue was significantly lower than that in HC decidual tissue. These data further elucidate the relationship between SHP2 and RM.

## Discussion

Decidualization is essential for embryo implantation and pregnancy maintenance (Zhang *et al.* 2022*a*). Decidualized ESCs can be used as a sensor of embryo quality after implantation (Sandra *et al.* 2011). Normal decidualization rejects the implantation of low-quality embryos and leads to rapid death through shedding similar to menstruation, which is a natural embryo selection mechanism that limits the mother’s investment in pregnancy with impaired development (Weimar *et al.* 2012). Abnormal decidualization allows decidual implantation without selectivity but cannot maintain pregnancy (Teklenburg *et al.* 2010). In the present study, we proved that the expression of SHP2 increased significantly during decidualization induction, and the same data were obtained in tissue samples, indicating that SHP2 may play an important role in regulating the biological behavior of decidualization.

SHP2 is a nonreceptor protein tyrosine phosphatase encoded by the protein tyrosine phosphatase nonreceptor type 11 gene (PTPN11) (Kong & Long 2022). SHP2 is widely expressed and plays a key role in cell growth, survival, proliferation, differentiation, and migration (Idrees *et al.* 2020). In most cases, SHP2 is necessary for the Ras–ERK and PI3K–Akt pathways and plays a dynamic role in gametogenesis, embryo maturation, and embryonic development (Idrees *et al.* 2020). SHP2 upregulation enhances Ras-ERK signaling, thereby inhibiting LIF receptors and thus downregulating JAK–STAT3 signaling, ultimately promoting cell differentiation and specialization (Cha & Park 2010). In addition, SHP2 plays an important role in several types of stem cell proliferation by simultaneously activating Ras–ERK and JAK–STAT signaling (Shen *et al.* 2020). SHP2 regulates adhesion proteins through focal adhesion kinase (FAK) to maintain cell–cell interactions/adhesion, especially in the blood testicular gap junction (Puri & Walker 2013). In addition, the tyrosine phosphatase SHP2 increases cell movement through SFKs (Hartman *et al.* 2013). In addition to playing an important role in embryonic development, SHP2 is also crucial for early embryo implantation (Gu *et al.* 2021). The expression of SHP2 is essential for the proliferation and differentiation of trophoblast stem cells, which are special placental cells that are able to invade maternal tissue (Forbes *et al.* 2009). A recent study found that uterine-specific SHP2 deletion reduced ER-a transcription and inhibited the expression of uterine-specific PR, which is essential for embryo implantation (Ran *et al.* 2017). Knockout of SHP2 from the embryo or the mother’s uterus completely inhibited implantation (Yang *et al.* 2006). Another study showed that SHP2 plays a key role in matrix decidualization by mediating a variety of signaling pathways in uterine stromal cells (Cheng *et al.* 2022). RMC-4550 is a better potent and selective allosteric inhibitor of SHP2, which was highly selective for full-length SHP2 over other phosphatase, kinase, and safety pharmacology targets (Nichols *et al.* 2018, Bracht *et al.* 2019, Wang *et al.* 2020). Here, we found that SHP2 blockade can affect the process of decidualization by not only affecting the expression of decidualization marker factors but also the morphological reprogramming of decidualized cells. The biological behavior of decidualization in pregnant mice treated with the SHP2 inhibitor RMC-4550 was also significantly inhibited, and the expression of progesterone downstream target genes was significantly reduced. These results suggest that SHP2 plays an important role in maintaining pregnancy by regulating the process of decidualization.

PR signaling plays a key role in the establishment and maintenance of pregnancy (Cope & Monsivais 2022). Complete ablation of progesterone signaling *in vivo* will lead to pregnancy failure (Conneely *et al.* 2001). PR signaling mainly involves PR and progesterone (Nothnick 2022). PR is a member of the nuclear receptor family. In the absence of progesterone ligand, PR is located in the cytoplasm as an inactive protein complex with inhibitory heat shock protein (HSP) 90 and HSP70, immunoglobulin and other factors (Jee *et al.* 2021). After ligand binding, PR is phosphorylated, inhibits protein dissociation and receptor dimerization, translocates to the nucleus, and combines with target genes at the specific palindromic progesterone response element on active chromatin that is ready for transcription. At the same time, to increase the complexity of the progesterone signal, the activity of PR can also be regulated by posttranslational modification, such as the phosphorylation of serine residues (mainly Ser294) in the N-terminal region of PR (Abdel-Hafiz & Horwitz 2014, Dwyer *et al.* 2020). This modification also plays an important role in regulating progesterone function in endometrial cells because it can stabilize the receptor and change its transcriptional activity, which can be induced when ligands are added (Cope & Monsivais 2022). Compared with the HC group, the phosphorylation of the Ser294 residue of PR was significantly reduced in ESCs of RM patients (Hosseinirad *et al.* 2021). Among them, the MAPK signaling pathway plays an important regulatory role in the phosphorylation of PR, while SHP2 is necessary for the molecular processes of Ras–ERK (Eaton *et al.* 2013). Activation of SHP2 is a crucial event for the induction of the ERK cascade (Pao *et al.* 2007, Rocha-Resende *et al.* 2017, Fedele *et al.* 2018, Anderson & Mazzoccoli 2019). SHP2 regulates the cellular morphological changes that occur through the ERK activation pathway and the small GTPases RhoA and Rac1 (Nakaoka *et al.* 2010). In this study, we found that the SHP2 inhibitor RMC-4550 could inhibit the nuclear translocation of PR and the expression level of PR downstream target genes, and the use of the SHP2 inhibitor RMC-4550 could inhibit the expression level of PR downstream target genes in decidual tissue of pregnant mice. In this study, we evaluated the effect of RMC-4550 on decidualization in mice by intraperitoneal injection, but it has some limitations, the most important of which is that intraperitoneal injection may cause systemic effects, followed by the possibility of wrong injection thus leading to complications such as perforation and bleeding of abdominal organs. There are also studies that have used uterine horn injections to perform experiments, but there are limitations to this approach. First, uterine horn injections require exposure to a larger surgical wound, which is more prone to infection; secondly, the drugs we use need to be dissolved using corn oil, and injecting corn oil in the uterine horn can induce spontaneous decidualization in the mouse uterus, which affects the experiment. Thus, additional studies are required to elucidate the effect of SHP2 inhibitors on endometrial decidualization in mice.

We further found that the SHP2 inhibitor RMC-4550 could inhibit the nuclear translocation of PR by inhibiting the phosphorylation of ERK. These results suggest that SHP2 participates in decidualization by regulating the ERK signaling pathway to maintain the normal nuclear localization of PR. These findings provide effective evidence for the future production of drugs that can either enhance or inhibit PR function; drugs that can enhance PR function would include drugs that can promote its entry into the nucleus to potentially treat RM and that may have clinical application in other progesterone-related conditions, such as endometriosis, while the development of drugs that can inhibit the function of PR may be useful in breast cancer and endometrial cancer. However, whether SHP2 regulates PR function directly or indirectly through other signaling pathways requires further experiments.

RM is a common and difficult reproductive disorder (Ewington *et al.* 2019). However, less than 50% of RM couples are diagnosed with a definite cause (Toth *et al.* 2010). Our experimental results demonstrated SHP2 was upregulated in decidualization and the SHP2 inhibitor RMC-4550 and shRNA-mediated SHP2 reduction attenuated decidualization *in vitro* and *in vivo*. Since RM is frequently associated with poor decidualization, we also examined its expression in RM decidua and found that SHP2 expression is blocked in RM, while the reason why SHP2 is suppressed in RM needs further exploration, as it is possibly related to factors such as hormone levels *in vivo* or the genetic background of the patients. There are currently no clear markers of miscarriage, and SHP2 downregulation holds promise as one of the markers of RM in the future. In recent years, an increasing number of studies have elucidated the significant characteristics of SHP2 in the signaling pathway of crucial events during tumorigenesis. Furthermore, SHP2 has important functions in multiple cell types involved in the tumor microenvironment (Song *et al.* 2021). More importantly, it was reported that SHP2 contributes to the pathomechanism of endometriosis (Huang *et al.* 2020). Since the pathophysiology of RM shows similar characteristics with endometriosis processes and endometriosis is associated with early pregnancy losses and possibly RM. In the cases of endometriosis, other factors associated with the disease also are susceptible to causing miscarriages and possibly RM (Pirtea *et al.* 2021). The increased expression of SHP2 might imply similar functions in RM. Although SHP2 has not been reported in RM, the SHP2 regulated RAS/ERK MAPK cascade, PI3K, and JAK/STAT signaling, which have been notably confirmed in abnormal signaling of RM and these signaling pathways are being regarded as therapeutic potential targets (Yotova *et al.* 2011, McKinnon *et al.* 2016). Therefore, we consider SHP2 to be ‘promising’ potential biomarker of RM. Moreover, our work also suggests a potential SHP2-based therapeutic strategy for RM, and more studies are needed in the future to evaluate other functions that are disrupted by SHP2 dysfunction.

**Figure 7:**
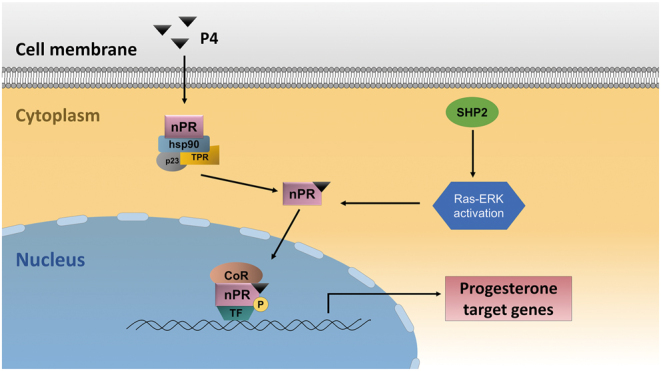
Illustrative model of uterine SHP2 maintaining PR function during decidualization. In the absence of progesterone ligands, PR and chaperone complexes form inhibitory proteins, including heat shock protein 90 (hsp90), p23 and cochaperones containing tetratricopeptide repeats (TPRs). The binding of progesterone activates PR by inducing conformational changes, phosphorylation, nuclear translocation, and recruitment of a series of coactivators (CoA) to regulate target gene transcription. SHP2 participates in decidualization by activating Ras–ERK to maintain the normal nuclear localization of PR and the expression of downstream target genes. CoR, coregulator; P4, progesterone; PR, progesterone receptor; SHP2, Src homology-2 domain-containing protein tyrosine phosphatase-2; TF, transcription factor.

Overall, our experimental results suggested upregulation of SHP2 is part of the decidualization program and that upregulated SHP2 is involved in decidualization by maintaining the normal nuclear localization of PR through the ERK signaling pathway (Fig. 7). This pathway serves as a bridge linking impaired decidualization resulting from SHP2 downregulation and the occurrence of miscarriage, providing a new scientific and clinical understanding of the occurrence of unexplained miscarriage.

## Declaration of interest

The authors declare that there is no conflict of interest that could be perceived as prejudicing the impartiality of the research reported.

## Funding

This work was supported by the National Natural Science Foundation of Chinahttp://dx.doi.org/10.13039/501100001809 (NSFC, Grant No. 82000150 and 82202579), Shenzhen Science and Technology Innovation Commission (JCYJ20190814164601648, JCYJ20210324123003009, JCYJ20220530144814032 and JCYJ20210324123210028), Guangdong Provincial Key Laboratory of Digestive Cancer Research (No. 2021B1212040006), Guangzhou industry university research collaborative innovation major project (No. 2016201604030009).

## Author contribution statement

Lin Chen and Weijie Zhao designed the research, performed the experiments and wrote the paper. Mengxiong Li assisted with the collection of human samples and assisted with the experiments. Yazhu Yang, Chengzi Tian, Dengyang Zhang, Zhiguang Chang and Yunzhe Zhang assisted with the experiments. Zhizhuang Joe Zhao, Yun Chen and Lin Ma assisted with the data analysis. All authors discussed the results and commented on the manuscript.
